# Significance of gender in the attitude towards doctor-patient communication in medical students and physicians

**DOI:** 10.1007/s00508-016-1054-1

**Published:** 2016-08-11

**Authors:** Henriette Löffler-Stastka, Tamara Seitz, Sabrina Billeth, Barbara Pastner, Ingrid Preusche, Charles Seidman

**Affiliations:** 1 Klinik für Psychoanalyse und Psychotherapie und Postgraduate Education and Training Center, Medizinische Universität Wien, Wien, Österreich; 2 SMZ Süd – KFJ, Medizinische Universität Wien, Wien, Österreich; 3 Klinik St. Pölten, St. Pölten, Österreich; 4 Vertinärmedizinische Universität Wien, Wien, Österreich; 5 Emory University, Atlanta, USA

**Keywords:** Doctor-patient communication, Therapeutic attitude, Gender dyads, Medical students, Communicative skills

## Abstract

**Background:**

Gender-specific differences in the attitudes towards doctor-patient communication among medical students and physicians were assessed.

**Methods:**

A total of 150 medical students and 51 physicians from different departments took part in the study. The association, attitude and experiences regarding doctor-patient communication were assessed with a series of tools and questionnaires.

**Results:**

Female doctors and students tended to describe the doctor-patient communication with positive attributes, such as *“helpful”, “sentimental”, “voluble”, “sociable”, “gentle”, “yielding”* and “*peaceful”*. Male students and physicians, on the other hand, described doctor-patient communication as *“overbearing”, “robust”* and *“inhibited”*. The most frequent associations females had with the term doctor-patient communication were *“empathy”, “confidence”, “openess*”, while the most frequent association of the male colleagues was *“medical history”.* Female doctors reported speaking about the psychosocial situation of the patient significantly more often and believed in higher patient satisfaction by sharing more information. Furthermore, they reported having longer conversations with a more equal partnership than their male colleagues. Compared to male students, female students were willing to take part in training their communication skills more often and had more interest in research about doctor-patient communication. Male medical students reported self-doubt during conversations with female patients, while one third of the male physicians talked about “*the power over the patient*”.

**Conclusions:**

This study indicates a gender-dependent communication style influenced by stereotypes. At the establishment of communication training these differences should be taken into account, especially to strengthen male communication skills and improve their attitudes.

## Introduction

Effective doctor-patient communication is essential for high-quality medicine [1]. Physicians’ communication skills influence a variety of factors in clinical practice and a multitude of studies have documented that effective doctor-patient communication improves patient satisfaction, patient recall and understanding, adherence to treatment and outcome and including symptom reduction. Furthermore, effective communication can also improve the physician’s job-satisfaction and well-being [2]. These skills have been shown to be teachable. Several (international) consensus statements have stressed the importance of training communication skills in medical curricula and defined competencies and educational objectives medical students should achieve by the end of their medical studies [3–5]. These educational objectives are comprised of training attitudes (facilitated e. g. by feedback or reflection tasks) as well as communication skills (e. g. communication style). Most interestingly, to the best of our knowledge, gender differences in communication skills were not defined as teachable educational objectives by experts until recently. Dielissen et al. [6] closed this gap by developing three gender criteria covering content skills (i. e. gathering and giving information), processing skills (verbal and non-verbal gender-sensitive communication behavior) and perceptual skills (handling emotions and gender-sensitive attitudes). These efforts are further continued in research approaches focusing on the influence of gender dyads on doctor-patient communication outcomes. In their systematic review, Sandhu et al. [7] highlighted the influence of gender differences on agendas elicited from patients, talk content, communication style, non-verbal communication, exhibition of power and status and duration of consultation; however, this review was based on studies using assessment tools that focused on coding behavioral outcomes of observed doctor patient communications. We do not know, however, how physicians’ and medical students’ attitudes towards doctor-patient communication itself are shaped by gender differences in doctor-patient relationships. A study focusing on these attitudes seems to be an important addendum to the existing body of research although attitudes do not automatically translate into corresponding behavior. Thus our study sought to answer the question of whether there is a gender-specific difference in physician attitudes towards communication with patients. The assessment was performed by self-rating and evaluation of questionnaires. The study was conducted with both physicians and medical students in order to gain insights into the development of gender-specific attitudes on doctor-patient communication skills.

## Participants, materials and methods

### Student sample

A total of 178 medical students in the seventh semester of the medical curriculum at the Medical University of Vienna gave informed consent to participate in the study. The questionnaires for the study were distributed to the students at the end of the compulsory lessons in psychotherapy within the framework of the university course lasting for 5 weeks, entitled “Psychic functions in health and illness”. The response rate was 88 %. In all 150 questionnaires were returned by the medical students (*n*_male_ = 65, *n*_female_ = 85, mean age 23.38 ± 3.05 years, min. 21 years, max. 39 years).

### Physician sample

A total of 34 female and 17 male physicians working in different departments (neurology *n* = 22, internal medicine *n* = 18 and gynecology *n* = 11) at a general hospital with a mandate to provide care participated in this study after giving informed consent. The mean age of the participants at the time of the survey was 36 years (±8.56 years, range 26–60 years). The response rate for the questionnaires varied from 62 % (questionnaire for evaluation of ward rounds) to 69 % (questionnaire on communication with the patient including semantic differential and open questions regarding the term doctor-patient communication).

## Assessment-Material

### Physicians’ and students’ connotations regarding doctor-patient communication

Physicians’ and students’ attitudes towards doctor-patient communication were evaluated using the 7‑point (range from −3 to +3) 25-item German standard semantic differential questionnaire [8] with the instruction *“The term doctor-patient communication appears to me like …”*. Participants were then given positively and negatively connoted descriptors and were asked to rate to what degree these descriptors adhered to their notions of doctor-patient communication. For example, the first positively or negatively connoted pair of descriptors is cheerful and sad: an answer of −3 means very cheerful, −2 rather cheerful and −1 slightly cheerful. For the negatively connoted descriptor, an answer of 1 means slightly sad, 2 rather sad and 3 very sad, where 0 is neutral. In contrast to questionnaires with self-rating the usage of this open and indirect instrument has several advantages as it avoids unintended effects of social desirability or inaccurate self-assessment by gathering unconscious attitudes of the participants.

### Physicians’ and students’ associations regarding doctor-patient communication

The physician and student participants were asked to write down three associations regarding the term doctor-patient communication in their own words.

### Physicians’ and students’ attitudes towards the medical interview

For measuring the physicians’ and students’ communication skills, their attitude towards doctor-patient communication and their evaluation of personal education experiences regarding the extent and benefit thereof, the questionnaire “communication with the patient” [9] was used.

### Students’ attitudes and assumptions towards doctor-patient communication

The therapeutic attitude scale (TASC-2) developed by Sandell et al. [10] was adapted to fit the students’ experiences and knowledge by specifying the instruction to their known level of expertise. The term *doctor-patient communication* instead of the term *psychotherapeutic work* was used. Furthermore, the term *patient *in the instruction was adapted to fit our research question by referring either to a female patient or a male patient. The students were randomly assigned to fill out a questionnaire with an instruction regarding a male patient or a female patient. Students were asked to fill out the TASC-2 questionnaire by recalling a recent interview they had conducted in their clinical elective either with a female or male patient in which the patient’s medical history was recorded. The TASC-2 is composed of three sections (E1, E2 and F) [11]. The first section, *curative factors *(E1), consists of 33 items, rated on a 5-point Likert-type scale, ranging from 0 (*does not help at all*) to 4 (*helps a lot*). These items correspond to three scales that represent different attitudes towards or beliefs of curative factors in doctor-patient communication: adjustment, insight and kindness. High scores in “adjustment” (e. g. giving the patient concrete advice) means that the student considers adjustment of the (male or female) patient’s emotions and behavior to the environment as an important factor in doctor-patient communication. “Insight” (e. g. interpreting the patient’s body language) represents examples of attempts to uncover, clarify and interpret what is assumed to be hidden or repressed by the patient. High scores in “kindness” refer to a student’s rating of the importance of various ingredients in a friendly social relationship (e. g. consideration and taking good care).

The second section, *therapeutic style factors *(E2), consists of 31 items, rated on a 5-point Likert-type scale, ranging from 0 (*do not agree at all*) to 4 (*agree very much*). It refers to attitudes towards therapeutic approaches, including three scales (neutrality, supportiveness and self-doubt): High scores in “neutrality” (e. g. I do not answer personal questions from the patient) represent the student’s rating of the importance of keeping a personal distance from the patient. “Supportiveness” (e. g. It is important to show empathy with the patient’s problems) represents attempts to encourage, structure or question the patient. High scores in “self-doubt” refer to various ingredients of a friendly social relationship (e. g. I find it difficult to deal with the patient’s aggression) reflect mainly weak self-confidence.

The third section, *basic assumptions *(F), consists of 16 items rated on visual analogue scales, aims to rate basic assumptions about the nature of doctor-patient communication and the human mind, resulting in three scales: irrationality (i. e. beliefs about to what extent humans are in conscious control of themselves), artistry (i. e. is doctor patient-communication an art, drawn from the doctor’s talent, intuition and inspiration or a set of learnable skills?) and pessimism (i. e. are there optimistic beliefs about possibilities of development, change and understanding or not?) [11, 12].

### Physicians’ impressions of doctor-patient communication during ward rounds

To measure the personal impression of a doctor-patient communication encounter during their ward rounds, physicians filled out a questionnaire “Doctor-patient communication during ward rounds” [13], displaying sociodemographic data and the following questions: “How long was the encounter with the patient? Does the patient ask you questions? How often do you talk to each patient besides the ward rounds? How long are these communication encounters on average? Are the patients sufficiently informed regarding the medications’ effects and side-effects? Do you feel power over the patient?”

## Statistical methods

All statistical analyses were conducted using SPSS, version 17 (IBM, Inc., Armonk, NY), applying a comparison-wise significance level of 5 %. Data about sex, age and gender were extracted using descriptive statistics. A *t*-test was used to test differences between male and female participants. For analyzing the effect of gender dyad differences ANOVA was used. In the case of heterogeneity of variances the Mann-Whitney U‑test and Kruskal-Wallis test were used. The participants’ associations related to doctor-patient communication were ranked by frequency and clustered according to content corresponding to the methods of qualitative content analysis [14]. Interrater reliability was analyzed by using Cohen’s kappa.

## Results

### Physicians’ and student’s connotations regarding doctor-patient communication

The connotation analyses (semantic differential) revealed a gender stereotype rating behavior: statistical differences among female (*n* = 85) and male students (*n* = 65) occured for the items “helpful-selfish”, “cool-sentimental”, “voluble-withheld”, “withdrawn-sociable” and “wild-gentle”. Female students tended to view doctor-patient communication more often as “helpful” (*t* = 2.68, *p* = 0.008), “sentimental” (*t* = 2.72, *p* = 0.007), “voluble” (*t* = 2.47, *p* = 0.015), sociable” (*t* = 2.27, *p* = 0.025) and “gentle” (*t* = 2.28, *p* = 0.024) than male students. For three items (“soft-hard”, “libidinous-inhibited” and “obsequious-overbearing”) heterogeneity of variance was evident; however, the Mann-Whitney U‑test revealed only a significant difference in the category “obsequious-overbearing”: male students tended to rate doctor-patient communication as more “overbearing” than female students (*p* = 0.007).

Female physicians (*n* = 49) considered doctor-patient communication significantly more “peaceful” (*t* = 2.87, *p* = 0.006) and more “yielding” (*t* = 2.66, *p* = 0.010) than their male colleagues. Whereas male physicians (*n* = 49) rated doctor-patient communication significantly more towards the item “robust” (*t* = 2.548, *p* = 0.014), female physicians preferred a more balanced rating between the items (see Table 1).

**Table 1:** Positive and negative connotations regarding doctor-patient communication

Positive connotation	Negative connotation
Cheerful	Sad
Blurred	Clear
Strong	Weak
Generous	Thrifty
Passive	Active
Playful	Serious
Restrained	Open
Helpful	Selfish
Cool	Sentimental
Voluble	Withheld
Peaceful	Aggressive
Distracted	Sorted
Sober	Dreamy
Strict	Yielding
Withdrawn	Sociable
Robust	Tender
Happy	Sullen
Wild	Gentle
Stiff	Movable
Quiet	Loud
Fresh	Tired
Healthy	Sick
Soft	Hard
Libidinous	Inhibited
Obsequious	Overbearing

For three items (“cheerful-sad”, “libidinous-inhibited” and “obsequious-overbearing”) heterogeneity of variance was evident with a significant difference in the category “libidinous-inhibited”: male physicians rated doctor-patient communication as more “inhibited” than female physicians (*p* = 0.038).

### Physicians’ and students’ associations regarding doctor-patient communication

Associations of the term doctor-patient communication were ranked according to their frequency. Interrater reliability was analyzed by using ratings from two independent raters (S. B. and C. S.) and resulted in κ = 0.89. The most frequent associations that were given by students regardless of gender were “empathy” (22 %), “confidence” (5 %) and “openness” (3 %). There were no gender-specific differences in the answers. The leading association of female physicians was “confidence” (6 %), whereas the leading associations of male physicians were “medical history (anamnesis)” and “important” (both 6 %).

### Physicians’ and students’ attitudes towards the medical interview

Significantly more female than male students reported formal voluntary training in doctor-patient communication during their curriculum (*t* = 2.19, *p* = 0.030). Female students also showed significantly enhanced interest in research results regarding doctor-patient communication than their male colleagues (*p* = 0.002). They also viewed the treatment outcome as being significantly more influenced by the setting of the doctor-patient communication than male students (*p* = 0.017). Furthermore, significantly more female than students rated therapeutic success as being dependent on doctor-patient communication (*t* = 2.946; *p* = 0.0037). Regarding our physician sample, female physicians rated “empathy” during communication with the patient as more important (*p* = 0.027), included more psychosocial aspects of the patient for diagnostic purposes (*t* = 3.758 *p* = 0.0005) and significantly rated providing information as a key factor in enhancing patient satisfaction (*t* = 2.94; *p* = 0.0050).

### Students’ attitudes and assumptions towards doctor-patient communication

The results of the TASC-2 questionnaire revealed differing relevance of the factors within the student sample with some differences between the means for the four gender dyads. Female students scored higher on the curative factor “insight” regardless of the sex of the patient. Compared to other gender dyads, female students saw “kindness” (*p* = 0.027) for their male patients as an important curative factor. Male students communicating with female patients, scored significantly higher on the factor “self-doubt” (*p* = 0.05) than every other gender dyad, representing weak self-confidence in male students for this configuration in doctor-patient communication; however, as the factor “self-doubt” is described as being problematic [13] it seems better to see this latter interpretation as a tendency. All other curative factors, therapeutic style factors and factors based on assumptions towards doctor-patient communication showed no differences regarding the four gender dyads.

### Physicians’ impressions of doctor-patient communication during ward rounds

Female physicians reported having longer communication encounters with their patients during ward rounds: on average the communication lasted 4.94 min for the female physicians and 3.84 min for their male counterparts (df = 48, *t* = 2.9, *p* = 0.006). Furthermore, females provided additional time for doctor-patient communication besides ward rounds at an average of 1.8 times a day, in contrast to their male colleagues who reported an average of 1.06 times a day (df = 38, *t* = 2.02, *p* = 0.049). All of the female physicians answered the item “*do you feel power over the patient?*” with “*no*”. One third of the male physicians, on the other hand, reported feeling power over the patient (*p* = 0.002). No gender-specific differences were found regarding the frequency of patients’ questions, duration of communication encounters besides ward rounds and impressions that patients were sufficiently informed regarding the medications’ effects and side effects.

## Discussion

Doctors perform around 200,000 consultations during their professional career, making communication a core clinical skill that needs to be taught and learned. While previous studies investigating gender dyad effects tended to focus on actual communication behavior [15], it is important to scrutinize these attitudes to a greater extent as it can be seen as a prerequisite of the actual behavior. We identified students and physicians as a relevant study sample as we also thought to gain insight into the development of these gender-specific attitudes on communication skills.

In accordance with literature findings [7], the females in our study showed more positive attitudes towards doctor-patient communication than their male colleagues. These included the belief of its specific value, for example that good doctor-patient-communication leads to a better outcome and to a more successful therapy. Furthermore, female medical students reported taking part in individual and voluntary training of communication and showed interest in research regarding doctor-patient communication more often than their male counterparts. Female physicians showed a more patient-centered attitude, such as talking longer and more often with patients. Empathy, confidence, kindness and psychosocial aspects appeared to play a greater role for female physicians.

One of the most striking results is that one third of the male physicians displayed an attitude representing a paternalistic relationship. This seems to be in line with Ross-Lee [16] regarding the male physicians’ motives for their career, such as higher academic orientation, more interest for prestige, power and financial rewards.

Furthermore, the results of TASC-2 showed that male students’ attitudes towards communication with female patients revealed weak self-confidence. Research findings on the psychotherapeutic dyadic setting showed that dyads consisting of the same gender go more smoothly [17], which is mainly explained through psychosexual developmental arguments [18]. Due to a relatively minimal congenital prerequisite of reaction formation for social processes we rely on social forming processes as some kind of external stabilization [19]. Taking such socialization processes into account we have to point to the compellent forming based on social forming processes. As these considerations belong to theories concerning primary socialization processes, we would hypothesize that our results display a communication style influenced by socialized, cut and dried opinions and gender role stereotypes, as occupational secondary socialization often reactivates primary socialization factors. We would conclude that socialization processes, as well as subjectively important (unconscious) meanings and attributions play a specific role in communication processes. Further research, beginning at school age, is needed to clarify if teaching efforts are overshadowed by socialized gender stereotypes.

Limitations of this study are that we used different questionnaires for physicians (questionnaire related to ward rounds) and students (TASC-2) in order to try to display the respective working and educational environments better. This limits the significance of differences found between physicians and students; however, it is noticeable that students, especially female ones, more frequently mentioned social aspects, such as empathy and confidence, than physicians. Furthermore, male students reported having self-doubt while talking to a female patient. In contrast, one third of the male physicians felt power over the patients, which might be seen as a strategy to overcome these feelings of self-doubt. Another limitation is the small numbers in the sample of physicians. Nonetheless, our findings have several implications for doctor-patient communication. Firstly, gender-specific communication behavior and attitudes should be further emphasized in the context of research on doctor-patient encounters and the patients’ choice of physicians. Secondly, gender stereotyped communication is already established through attitudes of medical students and seems to be prolonged for practicing physicians This stresses the need for gender criteria in medical communication and education, as stated in the expert consensus paper by Dielissen et al. [6]. It might be especially necessary to strengthen male health providers’ positive attitudes towards doctor-patient communication. Thirdly, there are still open research questions regarding the specific gender dyad effects as seen from the patients’ perspective (e. g. using the methodology of grounded theory) or the concrete relation between gender stereotype attitudes, the intention to perform adequately and the actual performance (e. g. using the framework of the theory of planned behavior [20]) that need further investigations.
